# Three new species of the killifish genus *Melanorivulus* from the central Brazilian Cerrado savanna (Cyprinodontiformes, Aplocheilidae)

**DOI:** 10.3897/zookeys.645.10920

**Published:** 2017-01-12

**Authors:** Wilson J. E. M. Costa

**Affiliations:** 1 Laboratory of Systematics and Evolution of Teleost Fishes, Institute of Biology, Federal University of Rio de Janeiro, Caixa Postal 68049, CEP 21941-971, Rio de Janeiro, Brazil

**Keywords:** Biodiversity hotspot, morphology, osteology, systematics, taxonomy

## Abstract

Three new species are described from the Neotropical region comprising the Cerrado savannas of the central Brazilian plateaus, which is among the most important biodiversity centres in the world. These species are considered closely related to *Melanorivulus dapazi* from the same region, with which they share the presence of a rudimentary interarcual cartilage and a dark reddish brown distal margin on the male anal fin. The group comprising *Melanorivulus dapazi* and the three new species is here named as the *Melanorivulus dapazi* species group. *Melanorivulus ignescens* **sp. n.**, from the upper Rio Araguaia basin, is distinguished from all other species of the *Melanorivulus dapazi* group by the anal-fin colour pattern in males; *Melanorivulus flavipinnis* **sp. n.** and *Melanorivulus regularis* **sp. n.** from the Rio Paraguai basin are distinguished from all other congeners of the *Melanorivulus dapazi* group by the colour pattern of the caudal fin and number of scales in the longitudinal series, respectively. All the new species are further unambiguously diagnosed by unique combinations of morphological characters, including meristic and morphometric data, and colour patterns. This study reinforces the importance of using live colour patterns to diagnose species and species groups of the genus *Melanorivulus*, but also indicates that osteological characters may be informative for species diagnosis. This study confirms the high diversity of species of *Melanorivulus* in the central Brazilian Cerrado plateaus already reported in previous studies, indicating that endemic species are often restricted to short segments of a single river drainage.

## Introduction

The region comprising the Cerrado savannas of central Brazil has been considered among the most important biodiversity hotspots in the world (Myers et al. 2000), although many organisms endemic to this region were insufficiently sampled and poorly known until recent years (Costa et al. 2016). A typical component of the Cerrado fauna is the killifish genus *Melanorivulus* Costa, 2006, with species inhabiting the Veredas, a Cerrado ecosystem consisting of small streams running in shallow valleys, often exhibiting the buriti-palm *Mauritia flexuosa* along their banks (e.g., Costa 2007a; Oliveira et al. 2012). Probably as a consequence of small size, usually not surpassing 45 mm of total length, species of *Melanorivulus* occurring in this ecosystem were not represented in collections until recent years, with the great majority of the approximately 40 species occurring in the central Brazilian Cerrado being described only after 2005 (e.g., Costa 2012; Costa et al. 2016).

The greatest diversity among species of *Melanorivulus* endemic to the Cerrado is concentrated in the central-western Brazilian plateaus, which range in altitudes from 400 to 1,100 m above sea level (asl), in the Caiapó mountain range (Costa 2012). This area is drained by the upper tributaries of the Rio Araguaia, flowing north and belonging to the Amazonas–Tocantins river system, and the upper Paraguai and Paraná river basins, flowing southwest and south, respectively, and belonging to the Paraná–Paraguay–Uruguay river system. A total of 12 species have been recorded for this area, of which four are endemic to the Araguaia basin, one to the Paraguai basin, and seven to the Paraná basin (Costa 1989, 2005, 2006a, 2007a–b, 2008, 2012). During a recent expedition to this area, three new species were collected, one from the upper Araguaia basin and two from the Paraguai basin. All the three new species are considered to be closely related to *Melanorivulus dapazi*, endemic to the Paraguai basin, by all sharing a rudimentary interarcual cartilage and a dark reddish brown stripe on the distal margin of the anal fin in males (vs. interarcual cartilage well-developed and never a similar stripe on the anal-fin distal margin; see Discussion below). This assemblage is hereafter called the *Melanorivulus dapazi* species group and the three new species are herein described.

## Material and methods

Specimens were captured with small dip nets (40 × 30 cm) and were euthanized soon after collection. Representative live specimens were kept alive for nearly 24 hours, photographed, and then euthanized. Euthanasia was conducted in a buffered solution of tricaine methanesulfonate (MS-222) at a concentration of 250 mg/l, for a period of about 10 minutes, i.e., until opercular movements ceased. Specimens were fixed in formalin for a period of 10 days, and then transferred to 70% ethanol. Collections were made with permits provided by ICMBio (Instituto Chico Mendes de Conservação da Biodiversidade) and methods for euthanasia were approved by CEUA-CCS-UFRJ (Ethics Committee for Animal Use of Federal University of Rio de Janeiro; permit number: 01200.001568/2013-87). Material is deposited in Instituto de Biologia, Universidade Federal do Rio de Janeiro, Rio de Janeiro (UFRJ) and Coleção Ictiológica do Centro de Ciências Agrárias e Ambientais, Universidade Federal do Maranhão, Chapadinha (CICCAA).

Descriptions of colouration of living fish were based on photographs of both sides of individuals. Photographs were taken in small aquaria around 24 hours after collection. Additional direct observations were made with fish in small transparent plastic bottles just after collection. Measurements and counts follow Costa (1988). Measurements are presented as percentages of standard length (SL), except for those related to head morphology, which are expressed as percentages of head length. Fin-ray counts include all elements. Four specimens, two males and two males, were cleared and stained for osteological analysis using the methods presented in Taylor and Van Dyke (1985); the abbreviation C&S in lists of material indicates those specimens that were prepared for osteological examination. Terminology for osteological structures followed Costa (2006b), for frontal squamation Hoedeman (1958), and for cephalic neuromast series Costa (2001). Osteological characters used in species descriptions are those that show variability within *Melanorivulus* (e.g., Costa 2016). Herein, geographical localities involved terms popularly adopted in the local region to compose names of geographical accidents (e.g., rio, ribeirão) allowing more accurate identifications of localities in the field and avoiding common mistakes when tentatively translating them to English; following this reasoning, Rio Paraguai is used instead of Paraguay River. New species descriptions are listed according to their type localities, from north to south.

## Results

### Melanorivulus ignescens sp. n.

Taxon classificationAnimaliaCyprinodontiformesAplocheilidae

http://zoobank.org/01008AFD-8842-4DED-8E5F-4D7DF54463F9

**Figure 1.**
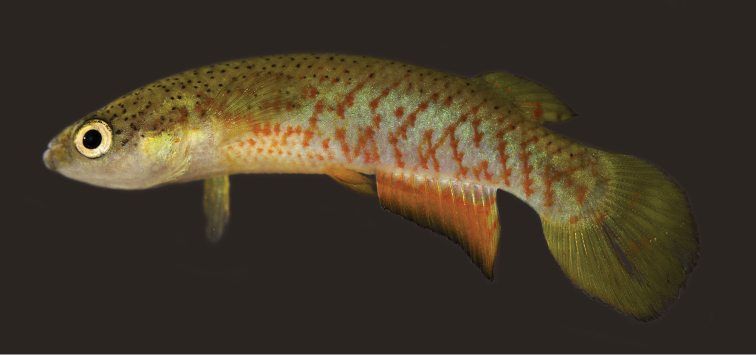
*Melanorivulus ignescens* sp. n., holotype, UFRJ 6875, male, 27.7 mm SL. Photograph by W.J.E.M. Costa.

**Figure 2.**
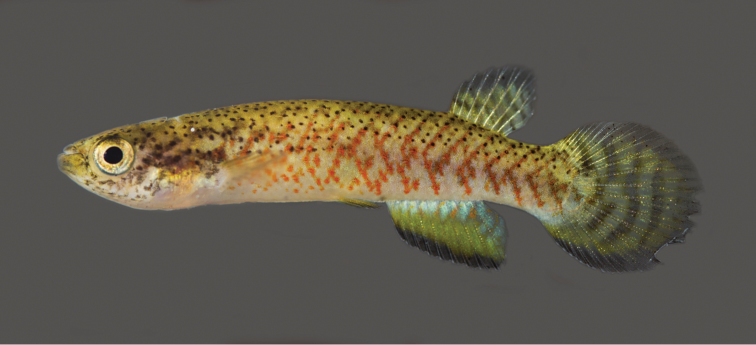
*Melanorivulus ignescens* sp. n., paratype, UFRJ 6876, female, 23.4 mm SL. Photograph by W.J.E.M. Costa.

Figs 1 , 2 , Table 1

#### Holotype.

UFRJ 6875, male, 27.7 mm SL; Brazil: Mato Grosso state: Guiratinga municipality: stream tributary to Rio Bandeira, Rio das Garças drainage, Rio Araguaia drainage, 16°21'54"S, 53°47'58"W, altitude approximately 520 m asl, road MT-270, approximately 3 km southwest of the village of Guiratinga; W. J. E. M. Costa et al., 11 August 2016.

#### Paratypes.

UFRJ 6876, 13 males, 15.8–25.5 mm SL, 18 females, 17.7–23.4 mm SL; UFRJ 6877, 2 males, 24.0–25.1 mm SL, 2 females, 22.4–23.4 mm SL (C&S); CICCAA00277, 1 male, 20.6 mm SL, 1 female, 18.6 mm SL; collected with holotype.

#### Diagnosis.

*Melanorivulus ignescens* is distinguished from all other species of the *Melanorivulus dapazi* group by having the anal fin, in adult males, bright reddish orange (vs. yellow in *Melanorivulus dapazi*, *Melanorivulus flavipinnis*, and *Melanorivulus regularis*). Also distinguished from all other congeners of the *Melanorivulus dapazi* group by the following combination of character states: 5–6 pelvic-fin rays (vs. 7 in *Melanorivulus dapazi* and *Melanorivulus regularis*); 29–31 scales in longitudinal series (vs. 35–37 in *Melanorivulus regularis*); female caudal spot inconspicuous in live fish (vs. conspicuous in *Melanorivulus dapazi* and *Melanorivulus regularis*); caudal fin, in males, without red bars and distinctive orange margin (vs. with red bars in *Melanorivulus regularis* and *Melanorivulus flavipinnis*, with broad bright orange band along the whole margin in *Melanorivulus dapazi*); in females, ventral surface of the head with dark grey spots, often forming short stripe on the chin (vs. without dark grey spots in *Melanorivulus dapazi*); caudal-fin short, its length 26.8–33.1% SL (vs. long, its length 34.1–38.7% SL in *Melanorivulus flavipinnis*). Also distinguished from all other species of the *Melanorivulus dapazi* group by having a constriction on the metapterygoid (vs. constriction absent).

#### Description.

Morphometric data appear in Table 1. Body slender, sub-cylindrical anteriorly, slightly deeper than wide, compressed posteriorly. Greatest body depth at vertical just in front of pelvic-fin base. Dorsal and ventral profiles of trunk almost straight to slightly convex in lateral view; dorsal and ventral profiles of caudal peduncle nearly straight. Head moderately wide, sub-triangular in lateral view, dorsal profile nearly straight, ventral profile convex. Jaws short, snout weakly pointed in lateral view.

**Table 1.** Morphometric data of *Melanorivulus ignescens*.

	holotype	paratypes	
	male	males (n = 9)	females (n = 6)
Standard length (mm)	27.7	20.1–25.5	21.4–24.7
**Percent of standard length**			
Body depth	22.1	21.3–22.3	20.5–22.8
Caudal peduncle depth	13.7	12.9–14.2	12.6–13.4
Pre-dorsal length	77.1	74.0–78.5	74.7–77.0
Pre-pelvic length	55.9	55.7–57.9	55.8–57.8
Length of dorsal-fin base	13.5	11.2–12.7	10.9–12.8
Length of anal-fin base	23.8	19.8–21.6	19.4–21.0
Caudal-fin length	33.1	29.9–32.9	26.8–32.0
Pectoral-fin length	21.4	19.1–21.5	18.0–20.6
Pelvic-fin length	10.8	9.4–11.1	8.2–9.7
Head length	27.6	27.2–30.9	27.2–30.0
**Percent of head length**			
Head depth	67.5	62.0–70.4	63.3–70.9
Head width	70.9	66.2–73.3	69.6–76.8
Snout length	13.2	10.9–13.5	12.1–14.1
Lower jaw length	21.6	15.9–19.5	15.3–21.4
Eye diameter	32.3	32.4–35.9	32.5–34.7

Dorsal and anal fins short, extremity slightly pointed in males, rounded in females. Caudal fin oval, slightly longer than deep. Pectoral fin rounded, posterior margin reaching vertical at 80–90% of length between pectoral-fin and pelvic-fin bases. Pelvic fin small, tip reaching between urogenital papilla and base of 1^st^ anal-fin ray in males, reaching between anus and urogenital papilla in females; pelvic-fin bases medially in close proximity. Dorsal-fin origin on vertical through base of 8^th^ anal-fin ray. Dorsal-fin rays 9–11; anal-fin rays 13–15; caudal-fin rays 30–31; pectoral-fin rays 13; pelvic-fin rays 5–6. No contact organs on fins.

Scales small, cycloid. Body and head entirely scaled, except anterior ventral surface of head. Body squamation extending over anterior 25% of caudal-fin base; no scales on dorsal and anal-fin bases. Frontal squamation F-patterned, rarely E-scale anteriorly overlapping F-scale; E-scales not overlapping medially; scales arranged in regular circular pattern around A-scale without exposed margins. Longitudinal series of scales 29–31; transverse series of scales 9; scale rows around caudal peduncle 16. No contact organs on scales. Cephalic neuromasts: supraorbital 3 + 3, parietal 1, anterior rostral 1, posterior rostral 1, infraorbital 1 + 11 + 1, preorbital 2, otic 1, post-otic 1–2, supratemporal 1, median opercular 1, ventral opercular 1, pre-opercular 2 + 4, mandibular 2–3 + 1, lateral mandibular 1, paramandibular 1.

Jaw teeth numerous, conical, irregularly arranged, outer teeth larger and slightly curved, inner teeth straight. Ventral process angulo-articular short, pointed. Ventral process of palatine short, slightly contacting quadrate. Mesopterygoid slender, posterior tip not reaching metapterygoid. Metapterygoid sub-rectangular, with constriction on middle portion. Dorsal portion of preopercle short and pointed, channel rudimentary. Basihyal sub-triangular, greatest width 50% of length; basihyal cartilage nearly 15% of total basihyal length. Six branchiostegal rays. Second pharyngobranchial teeth absent. Interarcual cartilage rudimentary. Fourth ceratobranchial teeth present, continuously arranged. Gill-rakers on first branchial arch 1 + 8. Vomerine teeth 2–4. Dermosphenotic present. Ventral process of posttemporal absent. Second proximal radial of dorsal fin between neural spines of 19^th^ and 21^st^ vertebrae, first proximal radial of anal fin between pleural ribs of 13^th^ and 15^th^ vertebrae. Total vertebrae 30–31.

#### Colouration.

***Males*.** Flank metallic green-blue to metallic light green, sometimes purple-blue above anal fin; oblique narrow orangish red bars irregularly arranged, often forming chevron-like marks anteriorly directed; horizontal rows of reddish orange dots on anteroventral part of flank, between bases of pectoral and pelvic fins; pale dark grey blotches on postorbital region mainly visible when fish is exposed to strong light. Dorsum light brown with black dots, venter white. Dorsal portion of head side light brown, ventral portion white; pale golden iridescence on opercular region. Jaws dark grey. Iris pale yellow, sometimes with dark brown bar on anterior and posterior portions. Dorsal fin light yellow with two or three oblique dark red bars on posterior portion of fin. Anal fin reddish orange in adult exemplars to yellowish orange in juveniles, basal portion bluish white, distal region becoming gradually dark red-brown, distal margin with high concentration of melanophores. Caudal fin light yellow, often with faint orange spots on middle portion; sometimes pale bluish posterior margin. Pectoral fin hyaline. Pelvic fin orange.

***Females*.** Side of trunk and head similar to males, but with paler colours. Ventral surface of head white, with dark grey spots often forming short stripe on chin. Dorsal fin pale yellow, with transverse series of grey spots; broad dark grey to black band on distal margin. Anal fin green-yellow, basal portion light blue with small red spots. Caudal fin pale yellow, with three or four dark grey bars, often interrupted; small black spot, smaller than pupil, on dorso-basal portion of fin overlapping anterior-most bar, more conspicuous in preserved specimens; broad dark grey to black band on whole fin margin.

#### Distribution.

**Figure 3.**
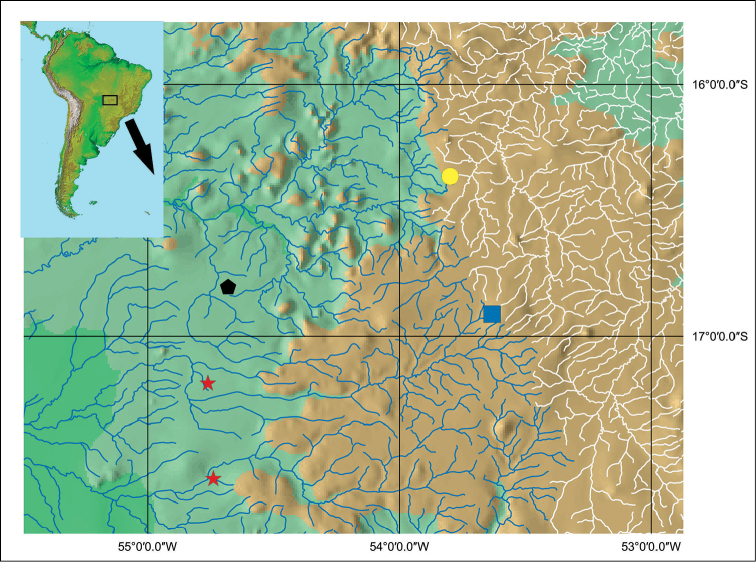
Geographical distribution of killifishes of the *Melanorivulus dapazi* species group. Yellow circle: *Melanorivulus ignescens*; black pentagon: *Melanorivulus flavipinnis*; blue square: *Melanorivulus regularis*; red star: *Melanorivulus dapazi*. Blue river drainage: Paraguai; white river drainage: Araguaia.

Known only from the type locality area, a small stream tributary to the Rio Bandeira, Rio das Garças drainage, upper Rio Araguaia basin, central Brazil, altitude approximately 520 m asl (Fig. 3).

#### Etymology.

From the Latin, *ignescens* (becoming inflamed), an allusion to the orange anal fin in males.

### Melanorivulus flavipinnis sp. n.

Taxon classificationAnimaliaCyprinodontiformesAplocheilidae

http://zoobank.org/6A6F3FA1-5867-4293-BCC6-D1782B35566C

**Figure 4.**
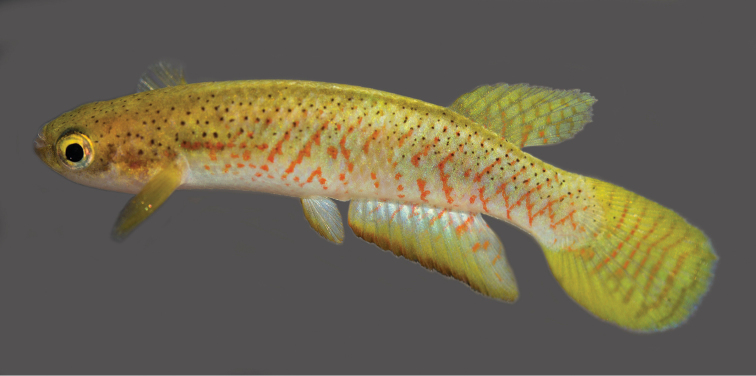
*Melanorivulus flavipinnis* sp. n., holotype, UFRJ 6881, male, 28.5 mm SL. Photograph by W.J.E.M. Costa.

**Figure 5.**
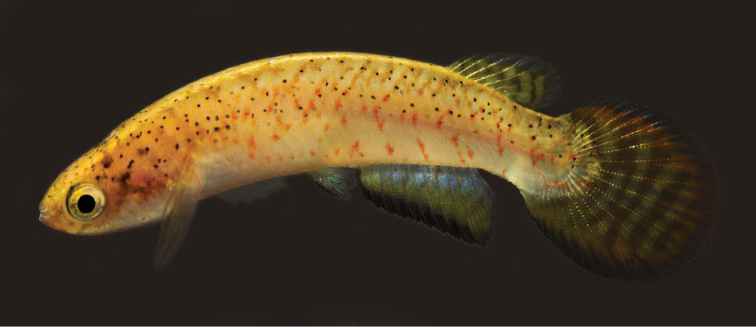
*Melanorivulus flavipinnis* sp. n., paratype, UFRJ 6882, female, 27.8 mm SL. Photograph by W.J.E.M. Costa.

Figs 4 , 5 , Table 2

#### Holotype.

UFRJ 6881, male, 28.5 mm SL; Brazil: Mato Grosso state: Rondonópolis municipality: stream tributary of Rio Anhumas, Rio São Lourenço drainage, Rio Paraguai basin, 16°48'16"S, 54°40'52"W, altitude approximately 420 m asl, road BR-070; W. J. E. M. Costa et al., 13 August 2016.

#### Paratypes.

UFRJ 6882, 2 males, 25.0–26.6 mm SL, 5 females, 22.3–39.4 mm SL; UFRJ 6883, 2 males, 22.5–25.6 mm SL, 2 females, 24.2–26.4 mm SL (C&S); CICCAA00279, 1 male, 25.7 mm SL, 1 female, 25.3 mm SL; collected with holotype.

#### Diagnosis.

*Melanorivulus flavipinnis* differs from all other species of the *Melanorivulus dapazi* group by the presence, in males, of seven or eight narrow red bars on the caudal fin, irregularly shaped and sometimes interconnected (vs. five or six dark red-brown regularly shaped and never interconnected bars in *Melanorivulus regularis*; four or fewer short rudimentary bars, sometimes absent, in *Melanorivulus dapazi*; bars always absent in *Melanorivulus ignescens*) and by the caudal fin, in females, being yellow on the middle portion and reddish orange on marginal region (vs. yellow to pale pink on the whole fin in the remaining species). Also distinguished from all other congeners of the *Melanorivulus dapazi* group by the following combination of character states: 5–6 pelvic-fin rays (vs. 7 in *Melanorivulus dapazi* and *Melanorivulus regularis*); 30–32 scales in longitudinal series (vs. 35–37 in *Melanorivulus regularis*); female caudal spot inconspicuous in live fish (vs. conspicuous in *Melanorivulus dapazi* and *Melanorivulus regularis*); caudal fin, in males, without distinctive orange margin (vs. with broad bright orange band along the whole margin in *Melanorivulus dapazi*); anal fin, in males, yellow (vs. reddish orange in *Melanorivulus ignescens*); in females, ventral surface of head with dark grey spots, often forming short stripe on chin (vs. without dark grey spots in *Melanorivulus dapazi*); caudal-fin long, its length 34.1–38.7% SL (vs. short, length 26.8–33.1% SL in *Melanorivulus ignescens*). Also distinguished from all other species of the *Melanorivulus dapazi* by the fourth ceratobranchial teeth arranged in two separate sections along the bone surface (vs. continuously arranged).

#### Description.

Morphometric data appear in Table 2. Body slender, sub-cylindrical anteriorly, slightly deeper than wide, compressed posteriorly. Greatest body depth at vertical just in front of pelvic-fin base. Dorsal and ventral profiles of trunk almost straight to slightly convex in lateral view; dorsal and ventral profiles of caudal peduncle nearly straight. Head moderately wide, sub-triangular in lateral view, dorsal profile nearly straight, ventral profile convex. Jaws short, snout weakly pointed in lateral view. Jaw teeth numerous, conical, irregularly arranged, outer teeth larger and slightly curved, inner teeth straight.

**Table 2.** Morphometric data of *Melanorivulus flavipinnis*.

	holotype	paratypes	
	male	males (n = 5)	females (n = 7)
Standard length (mm)	28.5	22.5–26.6	22.9–28.4
**Percent of standard length**			
Body depth	21.3	21.7–22.7	20.9–23.1
Caudal peduncle depth	13.3	13.2–14.3	12.8–14.1
Pre-dorsal length	72.3	73.0–76.2	73.8–76.4
Pre-pelvic length	53.7	52.6–55.1	53.0–56.8
Length of dorsal-fin base	14.6	11.2–13.7	10.4–13.4
Length of anal-fin base	24.7	21.4–25.2	20.6–23.3
Caudal-fin length	36.1	34.1–38.2	34.4–38.7
Pectoral-fin length	20.7	19.9–23.3	20.3–21.7
Pelvic-fin length	12.4	10.6–13.3	8.5–11.7
Head length	26.3	26.1–28.1	26.5–28.1
**Percent of head length**			
Head depth	67.0	65.5–71.3	66.0–73.5
Head width	69.4	67.4–72.7	70.9–76.2
Snout length	13.4	12.9–15.3	13.1–15.6
Lower jaw length	17.5	18.4–20.0	17.8–19.5
Eye diameter	33.4	32.3–35.2	28.8–35.8

Dorsal and anal fins short, tip slightly pointed in males, rounded in females. Caudal fin oval, longer than deep. Pectoral fin rounded, posterior margin reaching vertical at approximately 80–90% of length between pectoral-fin and pelvic-fin bases. Pelvic fin small, tip reaching between base of first and third anal-fin rays in males, reaching urogenital papilla in females; pelvic-fin bases medially in close proximity. Dorsal-fin origin on vertical through base of 8^th^ anal-fin ray. Dorsal-fin rays 9–10; anal-fin rays 14–15; caudal-fin rays 30–31; pectoral-fin rays 13; pelvic-fin rays 5–6. No contact organs on fins.

Scales small, cycloid. Body and head entirely scaled, except anterior ventral surface of head. Body squamation extending over anterior 25% of caudal-fin base; no scales on dorsal and anal-fin bases. Frontal squamation F-patterned, rarely E-scale anteriorly overlapping F-scale; E-scales not overlapping medially; scales arranged in regular circular pattern around A-scale without exposed margins. Longitudinal series of scales 30–32; transverse series of scales 8; scale rows around caudal peduncle 16. No contact organs on scales. Cephalic neuromasts: supraorbital 3 + 3, parietal 1, anterior rostral 1, posterior rostral 1, infraorbital 1 + 10–11 + 1, preorbital 2, otic 1, post-otic 1, supratemporal 1, median opercular 1, ventral opercular 1, pre-opercular 2 + 4, mandibular 2–3 + 1, lateral mandibular 1, paramandibular 1.

Jaw teeth numerous, conical, irregularly arranged, outer teeth larger and slightly curved, inner teeth straight. Ventral process angulo-articular short, pointed. Ventral process of palatine short, slightly contacting quadrate. Mesopterygoid slender, posterior tip not reaching metapterygoid. Metapterygoid sub-rectangular, with constriction on middle portion. Dorsal portion of preopercle short and pointed, channel rudimentary. Basihyal sub-triangular, greatest width about 50% of length; basihyal cartilage about 15–25% of total basihyal length. Six branchiostegal rays. Second pharyngobranchial teeth absent. Interarcual cartilage rudimentary. Fourth ceratobranchial teeth present, continuously arranged. Gill-rakers on first branchial arch 1 + 8. Vomerine teeth 2. Dermosphenotic present. Ventral process of posttemporal absent. Second proximal radial of dorsal fin between neural spines of 19^th^ and 21^st^ vertebrae, first proximal radial of anal fin between pleural ribs of 13^th^ and 15^th^ vertebrae. Total vertebrae 30–31.

#### Colouration.

***Males*.** Flank metallic green-blue to metallic light blue, sometimes purple-blue above anal fin; oblique narrow orangish red bars irregularly arranged, often forming chevron-like marks anteriorly directed; short light red stripe on humeral region; horizontal rows of reddish orange dots on antero-ventral part of flank, between bases of pectoral and pelvic fins; pale dark grey blotches on postorbital region mainly visible when fish is exposed to strong light. Dorsum light yellowish-brown with black dots, venter white. Dorsal portion of head side light brown, ventral portion white; pale golden iridescence on opercular region. Jaws dark grey. Iris pale yellow, sometimes with dark brown bar on anterior and posterior portions. Dorsal fin light yellow with seven or eight narrow oblique red bars, often forming reticulate pattern on distal portion of fin. Anal fin pale blue on its proximal half, with faint oblique red bars, light yellow in its distal half, distal region becoming gradually dark reddish brown on marginal border, distal margin with high concentration of melanophores. Caudal fin bright yellow, more intensely pigmented on dorsal and ventral portions, with seven or eight narrow red bars, irregularly shaped and sometimes interconnected. Pectoral fin yellowish hyaline. Pelvic fin light blue with orangish brown anterior margin.

***Females*.** Side of trunk and head similar to males, but with paler colours. Ventral surface of head white, with dark grey spots often forming short stripe on chin. Dorsal fin pale yellow, with oblique grey bars; broad dark grey to black band on distal margin. Anal fin green-yellow, basal portion light blue with small red spots. Caudal fin pale yellow on middle portion, reddish orange on marginal region, with five to seven dark grey bars, often interconnected; small black spot, smaller than pupil, on dorso-basal portion of fin overlapping anterior-most bar, conspicuous only in preserved specimens; broad dark grey to black band on whole fin margin.

#### Distribution.

Known only from the type locality, a small stream tributary to the Rio Anhumas, Rio São Lourenço drainage, Rio Paraguai basin, central Brazil, altitude approximately 420 m asl (Fig. 3).

#### Etymology.

From the Latin, *flavipinnis* (yellow fins), referring to the bright yellow colouration of the caudal fin in males.

### Melanorivulus regularis sp. n.

Taxon classificationAnimaliaCyprinodontiformesAplocheilidae

http://zoobank.org/6DE3B93B-9257-4557-B4C1-20B4D2BD05FE

Fig. 6 , Table 3

#### Holotype.

UFRJ 6878, male, 26.9 mm SL; Brazil: Mato Grosso state: Alto Graças municipality: Ribeirão da Sobra, upper Rio Itiquira drainage, Rio Paraguai basin, 16°54'41"S, 53°37'55"W, altitude approximately 750 m asl, road BR-364; W. J. E. M. Costa et al., 5 August 2016.

#### Paratypes.

UFRJ 6879, 4 males, 24.4–33.3 mm SL, 9 females, 22.3–33.8 mm SL; UFRJ 6880, 2 males, 25.3–31.2 mm SL, 2 females, 23.7–28.4 mm SL (C&S); CICCAA00278, 1 male, 24.7 mm SL, 1 female, 25.9 mm SL; collected with holotype.

#### Diagnosis.

*Melanorivulus regularis* is distinguished from all other species of the *Melanorivulus dapazi* group by the presence, in males, of five or six dark reddish brown, regularly shaped and never interconnected bars on the caudal fin (vs. seven or eight narrow red bars, irregularly shaped and sometimes interconnected in *Melanorivulus flavipinnis*; four or fewer short rudimentary bars, sometimes absent, in *Melanorivulus dapazi*; bars always absent in *Melanorivulus ignescens*). Also distinguished from all other congeners of the *Melanorivulus dapazi* group by the following combination of character states: 7 pelvic-fin rays (vs. 5–6 in *Melanorivulus flavipinnis* and *Melanorivulus ignescens*); 35–37 scales in longitudinal series (vs. 29–32 in *Melanorivulus flavipinnis* and *Melanorivulus ignescens*); caudal fin, in females, pale (vs. yellow on the middle portion and reddish orange on marginal region in *M flavipinnis*); female caudal spot conspicuous in live exemplars fish (vs. inconspicuous in *Melanorivulus flavipinnis* and *Melanorivulus ignescens*); caudal fin, in males, without distinctive orange margin (vs. with broad bright orange band along the whole margin in *Melanorivulus dapazi*); anal fin, in males, yellow (vs. reddish orange in *Melanorivulus ignescens*); in females, ventral surface of head with dark grey spots, often forming short stripe on chin (vs. without dark grey spots in *Melanorivulus dapazi*). Also distinguished from all other congeners of the *Melanorivulus dapazi* group by having 32 vertebrae (vs. 29–31)

#### Description.

Morphometric data appear in Table 3. Body slender, sub-cylindrical anteriorly, slightly deeper than wide, compressed posteriorly. Greatest body depth at vertical just in front of pelvic-fin base. Dorsal and ventral profiles of trunk almost straight to slightly convex in lateral view; dorsal and ventral profiles of caudal peduncle nearly straight. Head moderately wide, sub-triangular in lateral view, dorsal profile nearly straight, ventral profile convex. Jaws short, snout weakly pointed in lateral view. Jaw teeth numerous, conical, irregularly arranged, outer teeth larger and slightly curved, inner teeth straight.

**Figure 6.**
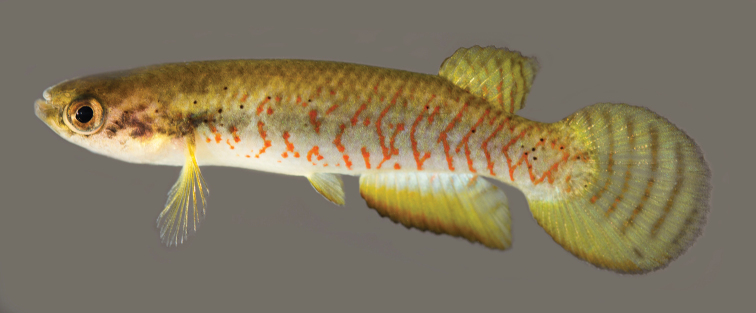
*Melanorivulus regularis* sp. n., holotype, UFRJ 6878, male, 26.9 mm SL. Photograph by W.J.E.M. Costa.

**Table 3.** Morphometric data of *Melanorivulus regularis*.

	holotype	paratypes	
	male	males (n = 7)	females (n = 7)
Standard length (mm)	26.9	24.4–33.3	23.7–33.8
**Percent of standard length**			
Body depth	23.2	21.9–22.6	21.6–23.0
Caudal peduncle depth	13.4	12.7–13.9	12.8–13.6
Pre-dorsal length	73.9	72.7–76.5	73.8–76.9
Pre-pelvic length	55.4	53.8–58.0	54.3–57.5
Length of dorsal-fin base	13.6	11.5–14.5	12.2–13.8
Length of anal-fin base	22.1	20.4–24.1	18.8–21.1
Caudal-fin length	34.1	32.7–33.7	31.7–34.9
Pectoral-fin length	21.4	20.0–22.2	19.2–21.5
Pelvic-fin length	10.7	9.6–12.4	8.4–10.2
Head length	27.2	25.3–27.5	24.9–27.2
**Percent of head length**			
Head depth	69.6	67.9–73.1	69.6–80.4
Head width	73.5	71.1–77.2	75.5–82.9
Snout length	15.2	13.6–17.1	13.4–16.2
Lower jaw length	20.1	16.6–20.7	20.1–23.7
Eye diameter	29.3	30.1–33.5	30.8–34.2

Dorsal and anal fins short, tip slightly pointed in males, rounded in females. Caudal fin oval, slightly longer than deep. Pectoral fin rounded, posterior margin reaching vertical at around 80% of length between pectoral-fin and pelvic-fin bases. Pelvic fin small, tip reaching between urogenital papilla and base of 1^st^ anal-fin ray in males, reaching anus in females; pelvic-fin bases medially in close proximity. Dorsal-fin origin on vertical through base of 8^th^ anal-fin ray. Dorsal-fin rays 10–11; anal-fin rays 14–15; caudal-fin rays 31–33; pectoral-fin rays 13–14; pelvic-fin rays 7. No contact organs on fins.

Scales small, cycloid. Body and head entirely scaled, except anterior ventral surface of head. Body squamation extending over anterior 25% of caudal-fin base; no scales on dorsal and anal-fin bases. Frontal squamation F-patterned, rarely E-scale anteriorly overlapping F-scale; E-scales not overlapping medially; scales arranged in regular circular pattern around A-scale without exposed margins. Longitudinal series of scales 35–37; transverse series of scales 9; scale rows around caudal peduncle 16. No contact organs on scales. Cephalic neuromasts: supraorbital 3 + 3, parietal 1, anterior rostral 1, posterior rostral 1, infraorbital 1 + 10–11 + 1, preorbital 1–2, otic 1, post-otic 1, supratemporal 1, median opercular 1, ventral opercular 1, pre-opercular 2 + 4, mandibular 2–3 + 1, lateral mandibular 1, paramandibular 1.

Jaw teeth numerous, conical, irregularly arranged, outer teeth larger and slightly curved, inner teeth straight. Ventral process angulo-articular short, pointed. Ventral process of palatine short, slightly contacting quadrate. Mesopterygoid slender, posterior tip not reaching metapterygoid. Metapterygoid sub-rectangular, with constriction on middle portion. Dorsal portion of preopercle short and pointed, channel rudimentary. Basihyal sub-triangular, greatest width near 55% of length; basihyal cartilage the 20% of total basihyal length. Six branchiostegal rays. Second pharyngobranchial teeth absent. Interarcual cartilage rudimentary. Fourth ceratobranchial teeth present, continuously arranged. Gill-rakers on first branchial arch 1 + 7–8. Vomerine teeth 2–5. Dermosphenotic present. Ventral process of posttemporal absent. Second proximal radial of dorsal fin between neural spines of 19^th^ and 21^st^ vertebrae, first proximal radial of anal fin between pleural ribs of 14^th^ and 15^th^ vertebrae. Total vertebrae 32.

#### Colouration.

***Males*.** Flank light metallic blue; oblique narrow orange-red bars irregularly arranged, often forming chevron-like marks anteriorly directed; horizontal rows of reddish orange dots on antero-ventral part of flank, between bases of pectoral and pelvic fins; dark brown pigmentation concentrated on postorbital, overlapped by black dots on superficial layer of skin. Dorsum light yellowish-grey, venter white. Dorsal portion of head side light brown, ventral portion white; pale golden iridescence on opercular region. Jaws dark grey. Iris pale yellow to pale brown. Dorsal fin pale yellow with four or five narrow red bars on posterior portion of fin. Anal fin orangish-yellow, basal portion white, posterior portion pale blue with two or three faint red oblique bars; distal region becoming gradually dark red-brown, distal margin with high concentration of melanophores. Caudal fin pale blue to pale yellow, with five or six dark red-brown regularly shaped bars, ventral portion light yellow without bars, ventral margin orangish-brown. Pectoral fin yellowish-hyaline. Pelvic fin pale blue with brown anterior margin.

***Females*.** Side of trunk and head similar to males, but with paler colours. Ventral surface of head white, with dark grey spots often forming short stripe on chin. Dorsal fin pale yellow, with three or four bars on posterior region; broad dark grey to black band on distal margin. Anal fin pale yellow, basal portion light blue. Caudal fin pale yellow, with four or five dark grey bars; small black spot, slightly smaller than pupil, on dorso-basal portion of fin; broad dark grey to black band on whole fin margin.

#### Distribution.

Known only from the type locality, Ribeirão da Sobra, an upper tributary of the Rio Itiquira, Rio Paraguai basin, central Brazil, in altitude about 750 m asl (Fig. 3).

#### Etymology.

From the Latin, *regularis* (regular), a reference to the caudal fin bars in males, regularly shaped and arranged on fin.

### Key to the species of the *Melanorivulus dapazi* group

**Table:** 

1	In females, ventral surface of head with dark grey spots, often forming short stripe on chin; in males, caudal fin never with broad bright orange band along entire margin	**2**
–	In females, ventral surface of head without dark grey spots; in males, caudal fin with broad bright orange band along margin	***Melanorivulus dapazi***
2	7 pelvic-fin rays; 35–37 scales in longitudinal series; female caudal spot conspicuous in live fish	***Melanorivulus regularis***
–	5–6 pelvic-fin rays; 29–32 scales in longitudinal series; female caudal spot inconspicuous in live fish	**3**
4	Caudal fin, in males, without bars; caudal fin, in females, pale yellow; anal fin, in males, bright red-orange; caudal-fin length 26.8–33.1% SL	***Melanorivulus ignescens***
–	Caudal fin, in males, with 7–8 red bars; caudal fin, in females, pale yellow on middle portion and orange on marginal portion; anal fin, in males, light yellow; caudal-fin length 34.1–38.7% SL	***Melanorivulus flavipinnis***

## Discussion

**Figure 7.**
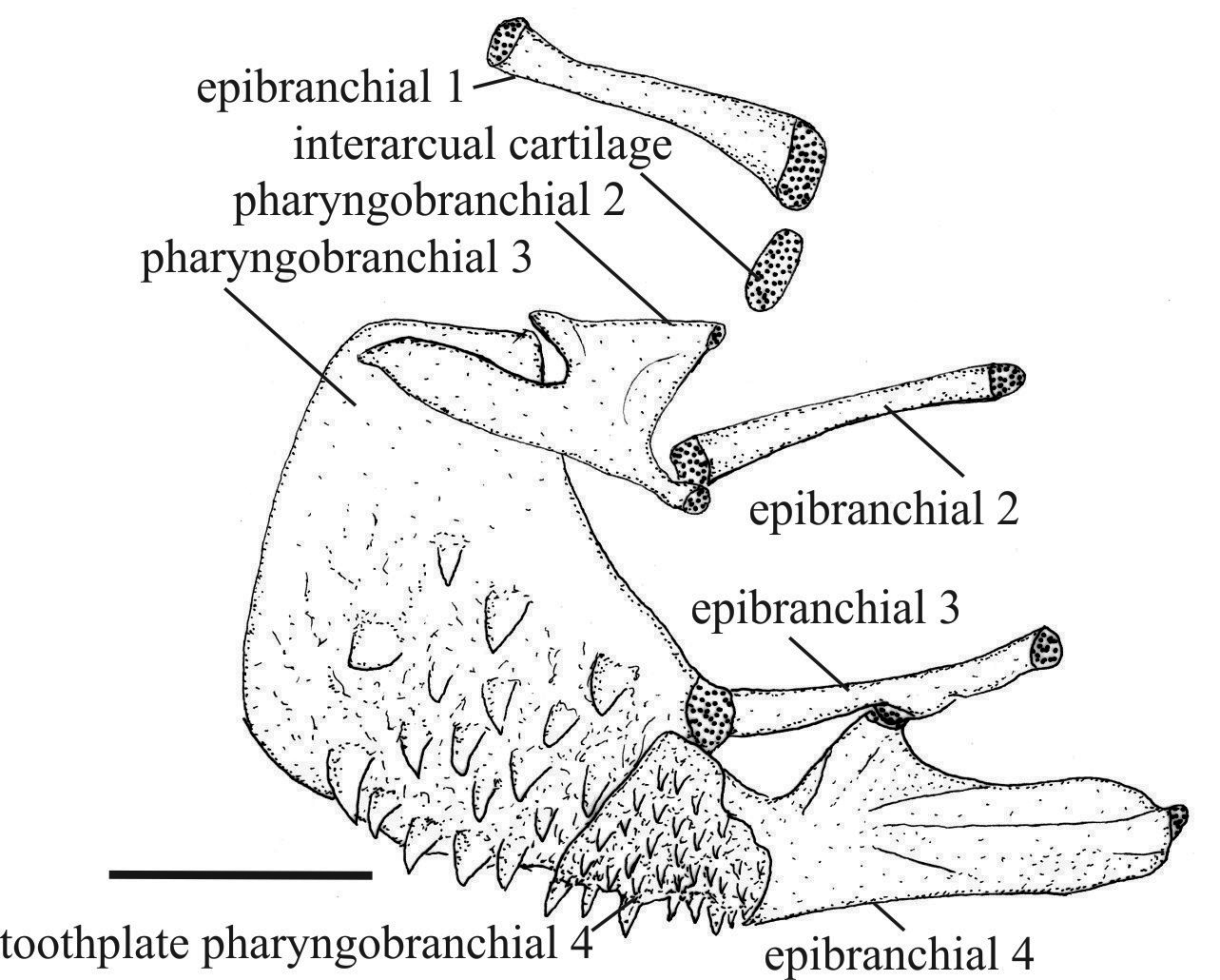
Dorsal branchial arches, left side, ventral view, of *Melanorivulus flavipinnis*, paratype, UFRJ 6883, 25.6 mm SL. Larger stippling indicates cartilage. Scale bar: 0.5 mm.

Morphological characters indicate that all three new species here described are more closely related to *Melanorivulus dapazi* than to other congeners, with these four species comprising the *Melanorivulus dapazi* group. In all species of this group, the interarcual cartilage is rudimentary, nearly equal in size to the adjacent cartilage at the tip of the first epibranchial (Fig. 7). In other species of *Melanorivulus*, the cartilage is well-developed, larger than first epibranchial cartilage, and around one fourth the length of the first epibranchial (e.g., Costa 2016: fig. 4). In addition, species of the *Melanorivulus dapazi* group share the presence of a dark red-brown distal margin on the male anal fin (Figs 1, 4, 6), a condition not found in other congeners. A molecular phylogeny of *Melanorivulus* (Costa et al. 2016) supports *Melanorivulus dapazi* as a sister group to a clade including species of the *Melanorivulus decoratus* group, but the three species here described were not included in that analysis. The *Melanorivulus decoratus* species group is diagnosed by the presence of five branchiostegal rays (vs. six) and a narrow basihyal, its width around 35% of the longitudinal length (vs. 45–60%). The *Melanorivulus decoratus* species group is comprised of three miniature species not surpassing 20 mm SL: *Melanorivulus atlanticus* Costa, Bragança & Ottoni, 2015, from the coastal plains of northeastern Brazil, *Melanorivulus decoratus* Costa, 1989, from the middle Rio São Francisco Basin, and *Melanorivulus jalapensis* Costa, 2010 from the middle Rio Tocantins drainage (Costa 1989, 2010; Costa et al. 2015).

**Figure 8.**
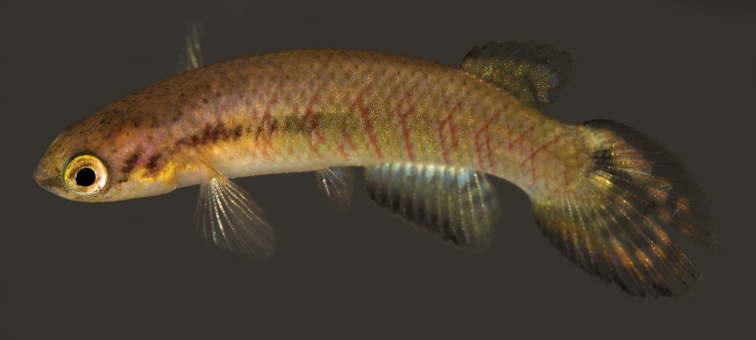
*Melanorivulus dapazi*, UFRJ 11203, female, 22.2 mm SL. Photograph by W.J.E.M. Costa.

**Figure 9.**
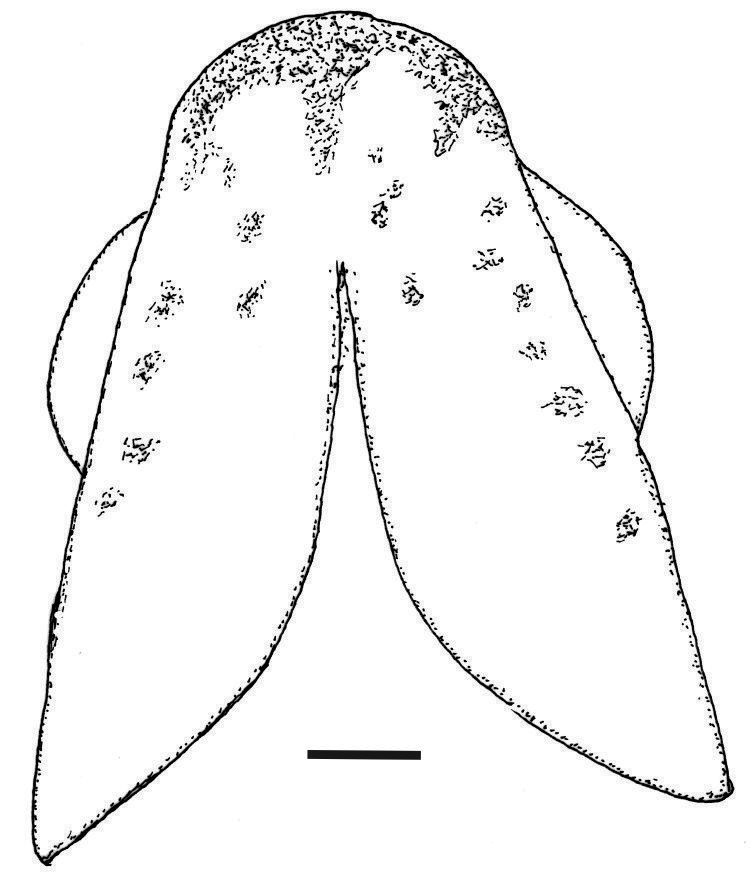
Diagrammatic representation of the colour pattern on the ventral surface of the head in females of *Melanorivulus regularis*, UFRJ 6879, 28.5 mm SL. Scale bar: 1 mm.

Relationships among species of the *Melanorivulus dapazi* group remain unclear. *Melanorivulus flavipinnis*, endemic to the Paraguai basin, is possibly more closely related to *Melanorivulus ignescens*, endemic to the Araguaia basin, than to *Melanorivulus dapazi* and *Melanorivulus regularis* that like *Melanorivulus flavipinnis* are endemic to the Paraguai basin. Among species of the *Melanorivulus dapazi* group, only in *Melanorivulus flavipinnis* and *Melanorivulus ignescens* there are five or six rays in the pelvic fin. In addition, in both species the spot on the basal portion of the female caudal fin is inconspicuous in live fish (Figs 2 and 5) and poorly visible in preserved specimens. In *Melanorivulus dapazi* and *Melanorivulus regularis*, there are seven pelvic-fin rays and the female caudal spot is conspicuous and delimited in live (Fig. 8) and preserved specimens, conditions considered plesiomorphic for *Melanorivulus* (Costa, 2016). The unique pigmentation pattern on the ventral surface of the head in females that is shared by *Melanorivulus flavipinnis*, *Melanorivulus ignescens*, and *Melanorivulus regularis* (Fig. 9), may be indicative of close relationships among these three species.

Costa (2016) discussed the importance of using live colour pattern characters to diagnose species and species groups of *Melanorivulus*, showing high congruence with molecular data. In that study, particular attention was given to patterns involving the caudal fin, which contained a high concentration of phylogenetically informative characters, useful to delimit most species of the *Melanorivulus zygonectes* group. Concordantly, the present study shows that colour patterns documented from live fish is an accurate tool to recognise species of the *Melanorivulus dapazi* group (see key for species identification above).

**Figure 10.**
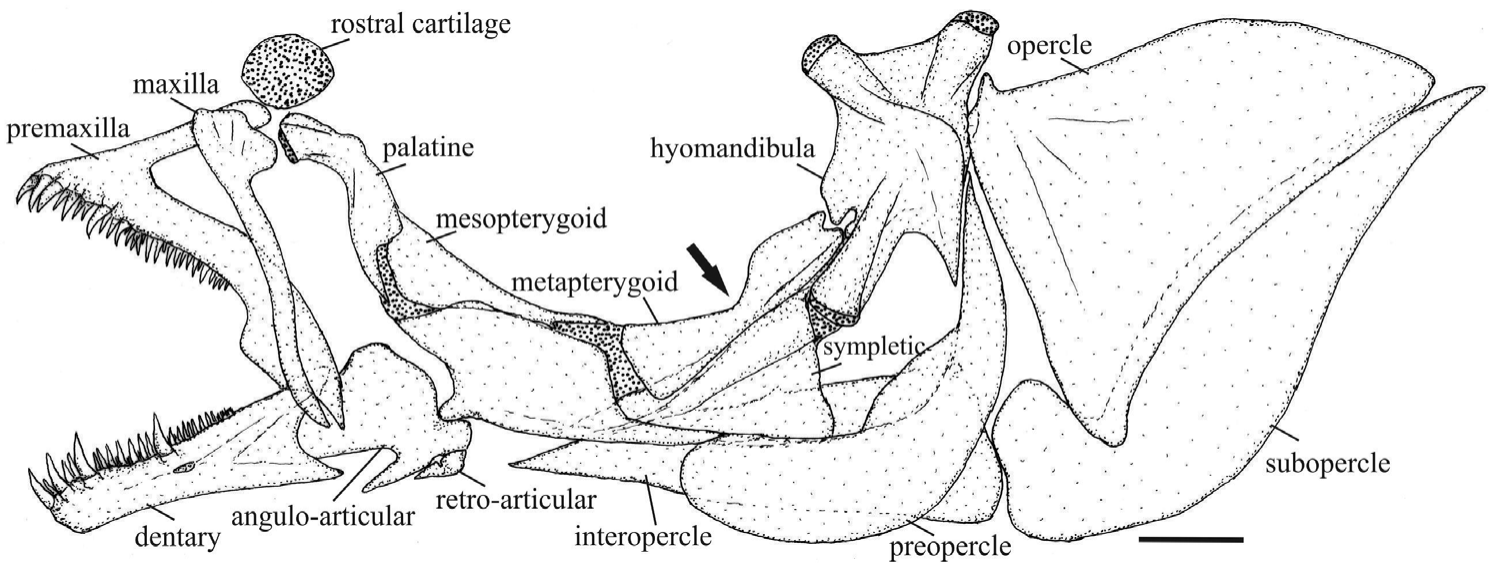
Jaws, jaw suspensorium and opercular apparatus, left side, lateral view, of *Melanorivulus ignescens*, paratype, UFRJ 6877, 25.1 mm SL. Larger stippling indicates cartilage. Scale bar: 0.5 mm.

**Figure 11.**
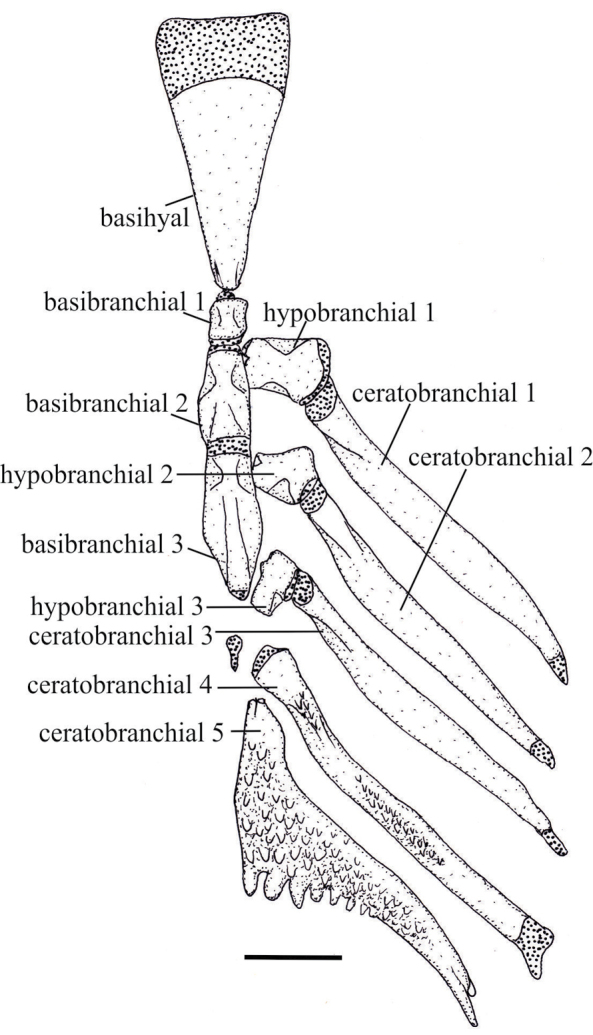
Basihyal and ventral branchial arches, right and median portion, dorsal view, of *Melanorivulus flavipinnis*, paratype, UFRJ 6883, 25.6 mm SL. Larger stippling indicates cartilage. Scale bar: 0.5 mm.

Osteological characters have been used to infer relationships among species groups of *Melanorivulus* and for diagnostic purposes (e.g., Costa 2016; this study). The present study shows that osteological characters may be also useful to diagnose single species. The unique shape of the metapterygoid recorded for *Melanorivulus ignescens*, with a constriction in its middle portion (Fig. 10), and the unique arrangement of teeth on the fourth ceratobranchial in *Melanorivulus flavipinnis*, exhibiting a median gap (Fig. 11), are not present in other congeners. In addition, *Melanorivulus regularis* differs from other species of the *Melanorivulus dapazi* group by having 32 vertebrae (vs. 29–31 in other species). Thus, although checking osteological characters in larger specimen samples is often not practicable, it is recommendable that osteology be included in taxonomical studies of *Melanorivulus* to complement species diagnoses.

Recent killifish inventories in the area of the central Brazilian plateaus drained by the upper tributaries of the Araguaia, Paraná and Paraguai river basins have revealed an unexpected high diversity of species of the genus *Melanorivulus* (e.g., Costa 2012). The present study confirms this high diversity, indicating once again that species inhabiting the region have small geographical ranges, often restricted to short segments of a single river drainage. For example, among species endemic to the Paraguai basin, *Melanorivulus regularis* was found in a single locality of the Rio Itiquira drainage, at approximately 750 m asl, whereas the present field survey indicated that at altitudes around 450 m asl of the same drainage, the only species found was *Melanorivulus dapazi*, which also occurs in similar altitudes of the neighbouring areas included in the Rio Correntes drainage (Costa 2005). On the other hand, *Melanorivulus flavipinnis* here described from the Rio São Lourenço drainage at approximately 420 m asl, is substituted by *Melanorivulus cyanopterus* (Costa, 2005), at altitudes of approximately 250 m asl (Costa 2005). The last species is a member of the *Melanorivulus punctatus* group, distantly related to the *Melanorivulus dapazi* group and geographically widespread in the lower Paraguai river basin (Costa et al. 2016). Recent studies with other vertebrates occurring in the Cerrado indicate that high species diversity in the region is correlated with the topographical reorganization during the Miocene, which generated geographical isolation of ancestral populations in plateaus and peripheral depressions (Prado et al. 2012; Guarnizo et al. 2016). This paleogeographical scenario may explain the present distribution of distinct species of *Melanorivulus* along different altitudinal zones of river drainages.

## Supplementary Material

XML Treatment for Melanorivulus ignescens

XML Treatment for Melanorivulus flavipinnis

XML Treatment for Melanorivulus regularis
